# Detection of serological biomarkers by proximity extension assay for detection of colorectal neoplasias in symptomatic individuals

**DOI:** 10.1186/1479-5876-11-253

**Published:** 2013-10-10

**Authors:** Stine Buch Thorsen, Martin Lundberg, Andrea Villablanca, Sarah Louise T Christensen, Kirstine Christensen Belling, Birgitte Sander Nielsen, Mick Knowles, Nick Gee, Hans Jørgen Nielsen, Nils Brünner, Ib Jarle Christensen, Simon Fredriksson, Jan Stenvang, Erika Assarsson

**Affiliations:** 1 Department of Veterinary Disease Biology, Faculty of Health and Medical Sciences, University of Copenhagen, Strandboulevarden 49, DK-2100, Copenhagen, Denmark; 2 Olink Bioscience, Uppsala Science Park, Dag Hammarskjölds väg 52B, SE-75237, Uppsala, Sweden; 3 Center for Biological Sequence Analysis, Department of Systems Biology, Technical University of Denmark DTU, DK-2800 Kgs., Lyngby, Denmark; 4 Innova Biosciences, Babraham Hall, Cambridge CB22 3AT, United Kingdom; 5 Department of Surgical Gastroenterology, Hvidovre Hospital, DK-2650, Hvidovre, Denmark; 6 The Finsen Laboratory, Rigshospitalet, Biotech Research and Innovation Center (BRIC), University of Copenhagen, DK-2200, Copenhagen, Denmark

**Keywords:** Colorectal cancer, Plasma, Biomarkers, Proximity ligation assay, Proximity extension assay

## Abstract

**Background:**

Although the potential of biomarkers to aid in early detection of colorectal cancer (CRC) is recognized and numerous biomarker candidates have been reported in the literature, to date only few molecular markers have been approved for daily clinical use.

**Methods:**

In order to improve the translation of biomarkers from the bench to clinical practice we initiated a biomarker study focusing on a novel technique, the proximity extension assay, with multiplexing capability and the possible additive effect obtained from biomarker panels. We performed a screening of 74 different biomarkers in plasma derived from a case–control sample set consisting of symptomatic individuals representing CRC patients, patients with adenoma, patients with non-neoplastic large bowel diseases and healthy individuals.

**Results:**

After statistical evaluation we found 12 significant indicators of CRC and the receiver operating characteristic (ROC) curve of Carcinoembryonic antigen (CEA), Transferrin Receptor-1 (TFRC), Macrophage migration inhibitory factor (MIF), Osteopontin (OPN/SPP1) and cancer antigen 242 (CA242) showed additive effect. This biomarker panel identified CRC patients with a sensitivity of 56% at 90% specificity and thus the performance is sufficiently high to further investigate this combination of five proteins as serological biomarkers for detection of CRC. Furthermore, when applying the indicators to identify early-stage CRC a combination of CEA, TFRC and CA242 resulted in a ROC curve with an area under the curve of 0.861.

**Conclusions:**

Five plasma protein biomarkers were found to be potential CRC discriminators and three of these were additionally found to be discriminators of early-stage CRC. These explorative data in symptomatic individuals demonstrates the feasibility of the multiplex proximity extension assay for screening of potential serological protein biomarkers and warrants independent analyses in a larger sample cohort, including asymptomatic individuals, to further validate the performances of our CRC biomarker panel.

## Introduction

Colorectal cancer (CRC) accounts for 608,700 deaths per year worldwide [1] which makes it one of the most common causes of cancer related deaths. Randomized clinical trials have demonstrated the value of population-based screening to reduce CRC-related mortality. In part, this can be ascribed to the detection of early-stage CRC with provision of timely treatment [2-4]. Therefore, there is a strong interest in the identification and clinical validation of new CRC biomarkers to be used for early detection of this disease.

Modalities as the Faecal Occult Blood Test (FOBT) and stool DNA (sDNA) tests are presently the only approved non-invasive screening tests available for detection of CRC in asymptomatic individuals. The performances of these tests have varied [4-6] and there is an immense problem with compliance [7,8]. In the results of a Danish study it was demonstrated that ≥60% compliance is a prerequisite in order to obtain successful effect of the screening [9]. A similar compliance problem in symptomatic individuals is not observed when using serological tests where the compliance is over 90% [10]. One potential serological CRC screening test is the Septin 9 (SEPT9) methylated DNA test which has demonstrated good test performance in a prospective screening study including nearly 8,000 asymptomatic individuals. However, the SEPT9 test still leaves about 33% of cancer patients undetected while the false-positive rate is 12% [11]. Furthermore, the technical platform could be improved as the SEPT9 test requires a large volume of plasma per test.

We addressed the clinical needs for a blood-based test by initiating a protein biomarker study evaluating 74 different protein biomarkers in plasma samples from case–control patient material consisting of symptomatic individuals represented by CRC patients, adenoma patients, patients with non-neoplastic large bowel diseases and healthy individuals. Presently, patients both with adenoma and CRC need further examination using endoscopy in order to evaluate the pathology of the neoplasm. Hence, it is not as important to discriminate between these two groups if the aim is to develop a pre-colonoscopy screening test. However, it is a technical challenge to measure proteins in plasma due to their biological complexity and a wide range in protein concentrations [12,13], a problem that is reflected in the general absence of serological CRC screening protein biomarkers being implemented in clinical settings [14,15]. Furthermore, in order to increase the success rate of a potential blood-based test, we found inspiration in a number of studies which demonstrated that higher discrimination power could be obtained by combining biomarkers [16-18]. However, aiming for a panel of biomarkers for a final test requires an assay with multiplexing capability, but without loss of technical sensitivity and specificity [19]. Moreover, low sample consumption and good assay performance in general is needed in order to facilitate high quality biomarker studies. We addressed these technical issues by applying the novel proximity extension assay (PEA) which is an improved version of a biomarker discovery tool with assay performance superior to the related Proximity Ligation Assay (PLA) in plasma samples [19].

To investigate the reason for the additive effect of our biomarker panel we evaluated the independence among the potential biomarkers as well as exploring their biological associations and interactions by performing network and pathway analysis.

In this study, we applied the novel PEA technique to identify five serological proteins that could discriminate between patients with colorectal neoplasias and control groups of healthy individuals and patients with other diseases. Furthermore, by focusing on the early stages of CRC in the statistical evaluation, we identified three proteins that are potential candidate biomarkers for early detection of CRC.

## Materials and methods

### Subjects and study design

Blood samples were obtained prospectively and consecutively prior to examination from individuals (aged 18+ years) undergoing sigmoidoscopy or colonoscopy, either following symptoms consistent with CRC or patients attending surveillance programs due to hereditary CRC (HNPCC and FAP). The study period was from 2003 to 2005 and samples were collected at six different centers in Denmark [10]. The case control study in the present manuscript is based on 4990 eligible individuals (including individuals with hereditary disposition, HNPCC or FAP) with 304 colorectal cancers (189 colon cancers and 115 rectal cancers, TNM stage I (n = 46), stage II (n = 88), stage III (n = 71) and stage IV (n = 72), remaining not staged), 10 other cancers, 923 adenomas, 1217 with non-neoplastic findings and 2536 with no findings [20]. According to the Helsinki II Declaration oral and written informed consent was obtained from each individual and the study was approved by The Regional Ethical Committee of Copenhagen and Frederiksberg, Denmark (KF 01-080/03). Subjects previously diagnosed with CRC and subjects unable to give informed consent were excluded from the study. Based on this study population, a case–control study was designed including 280 individuals representing four diagnostic groups of subjects. This study population was selected to test the potential of a panel of serological biomarkers to be used to detect CRC. First, 70 subjects with pathologically verified colorectal adenocarcinomas (25% rectal cancer and 75% colon cancer) were selected at random and subsequently, for each of these, a subject with histologically verified adenomatous changes (adenoma patients, n = 70) was randomly selected matching for age, gender and localization of the pathological finding. Then subjects with non-neoplasticlarge bowel disease (other diseases, n = 70) were randomly selected and matched as described for the adenomas, and lastly, subjects with no pathological findings by endoscopy and/or self-reported diseases or intake of medication (healthy individuals, n = 70) were selected in the same manner. The healthy individuals were characterized based on pre-endoscopy interview, data files from previous visits and results from colonoscopy/sigmoidoscopy as well as subsequent follow-up in persons with continuing symptoms as well. Individuals with other cancers were excluded from the group of healthy individuals. It appeared that three CRC patients, one adenoma patient and two patients with other diseases had a previous diagnosis of cancer (not CRC). These patients were included in the respective groups. The main clinical characteristics of subjects included in the study, except healthy individuals, are presented in Table 1.

**Table 1:** Patient characteristics

**Colorectal cancer patients**			**Patients with other disease**		**Adenoma patients**	
		**Subjects, n (%)**		**Subjects, n (%)**		**Subjects, n (%)**
*Gender*						
Female		36 (51)		36 (51)		36 (51)
Male		34 (49)		34 (49)		34 (49)
*Age group*						
40-49		3 (4)		3 (4)		3 (4)
50-59		7 (10)		7 (10)		7 (10)
60-69		16 (23)		16 (23)		16 (23)
70-79		24 (34)		24 (34)		24 (34)
80-99		20 (29)		20 (29)		20 (29)
					Adenoma	3 (4)
*Cancer stage*					Adenomateous lesion	1 (1)
*TNM*	*AJCC*				Serrated adenoma	1 (1)
T1, T2-N0-M0	I	7 (10)	Diverticular disease of colon NOS*	62 (89)	Mucous membrane	1 (1)
T3-N0-M0/T4-N0-M0	II	29 (41)	Diverticular disease of small intestine NOS*	3 (4)	Tubulovillous	15 (21)
T1, T2-N1-M0/T3, T4-N1-M0	III	15 (21)	Colitis NOS*	1 (1)	Tubular	40 (57)
Any T-N2-M0/Any T-Any N-M1	IV	14 (20)	Internal hemorrhoids NOS*	3 (4)	Villous	1 (1)
Not specified	NOS*	5 (7)	Haemorrhoids NOS*	1 (1)	NOS*	8 (11)

*NOS: Not otherwise specified.

Demonstrating the distribution of the different stages of cancer, the different types of diseases included in the group of patients with other diseases and the different types of adenomas included in the group of adenoma patients.

### Specimen characteristics

All blood samples were collected prior to large bowel endoscopy and consecutively according to a standard operating procedure (SOP) securing a high degree of uniformity among samples [21]. The plasma samples were prepared by collecting blood in EDTA tubes and placed on ice immediately after sampling. The samples were then centrifuged at 2500 *g* for 10 min at 4°C. After centrifugation the plasma was aspirated, aliquoted to new tubes and immediately hereafter stored at -80°C. From previous publications we found that more than six cycles of freeze-thaw could influence the data [22,23]. CEA and CA125 are more readily affected by long‒term frozen storage compared with frequent freezing–thawing [24] while such information is not yet available on CA242, TRFC, MIF, and OPN. Therefore, the samples used in the present study had undergone from one to four freeze-thaw cycles before analysis.

### Methods used for the preclinical exploratory study

The putative biomarkers were carefully selected for the biomarker screening by thorough literature searches exploiting databases as e.g. PubMed®, MEDLINE®, Google scholar®, UniProt®, GeneCards® and The human protein atlas (proteinatlas.org) [25]. The primary criteria included in the searches were proteins present in pathways involved in CRC, proteins involved in inflammation and cancer, proteins found in biomarker screening studies of CRC tumor tissue and general cancer markers. The different titles and abbreviations for each protein were carefully assessed. The technical construction of the assays was added to the criteria, as availability of appropriate antibodies for the proximity probes was needed. We applied the proximity extension assay (PEA), since its assay performance is superior to PLA when analyzing plasma samples [19]. In the present study, PEAs were constructed for the 74 different targets and an assay validation was performed with a focus on utilizing the PEA as a biomarker discovery tool.

### Assay methods in the assay validation study

#### Proximity probe preparation

The proximity probes in the 24-plex PEA setup were prepared by linking either pairs of matched monoclonal antibodies, or a single batch of affinity purified polyclonal antibody split in two, to either a 3′-hydroxyl free or a 5′-phosphate free 40-mer oligonucleotide (Additional file 1: Table S1 and Table S2). The proximity probes were generated by Innova Biosciences (Cambridge, UK) using their Lightning-LinkTM technology. Conjugation quality was analyzed by SDS-PAGE (data not shown). The 3′-hydroxyl or 5′-phosphate free oligonucleotides both comprise a unique flanking 20-bp sequence for primer binding during PCR and qPCR as well as a universal 20-bp sequence. Extension oligonucleotides contained a 40-bp sequence complimentary to each of the 5′ free oligonucleotides. In addition, each extension oligonucleotide contained a 16-bp universal sequence used to hybridize to the corresponding 3′ free oligonucleotide and unite the two oligonucleotides, and to generate a central 26 bp binding site for a Molecular Beacon. The hybridization was performed at room temperature for 20 min in a 4:1 oligo-to-probe ratio. The basic PEA protocol was performed by mixing 1 μL of PBS + 0.1% BSA ± antigen spike-in (antigens listed in Additional file 1: Table S3) or human EDTA plasma with 0.64 μL Probe mix [50 pM of each PEA probe pair, internal control standard spike-in mix (green fluorescent protein (GFP) (Vector Laboratories, USA) and extension oligonucleotide)] and 2.36 μL plasma dilution (Olink Bioscience, Sweden). The mix was incubated at 4°C overnight. Afterwards, tubes containing the 4 μL probed samples were transferred to a thermal cycler set at 37°C and 76 μL pre-extension mix (Olink Bioscience) was added, followed by incubation at 37°C for 5 min. Immediately after, 20 μl extension mix (Olink Bioscience) was added and the extension reactions were then run at 37°C for 20 min followed by a 10-min heat inactivation step at 80°C.

PEA was performed in multiplex using a 24-plex panel. All steps were performed as above, but using a set of the 24 unique probe oligo pairs, the 24 corresponding pre-amplification primers and the 24 unique primer pairs for qPCR detection. In the qPCR a universal Molecular Beacon (FAM-CCCGCTCGCTTATGCTACCGTGACCTGCGAATCCCGAGCGGG-DABSYL, Biomers) was used as detection system.

#### Screening procedure

*Pre-amplification* – was performed in PCR plates. A total volume of 25 μl by mixing 20 μl of the ligated product with 5 μL PCR mix [1x PCR buffer (Invitrogen, Denmark), 15 mM MgCl_2_ (Invitrogen), 1 mM dNTP (Invitrogen), 0.2 μM of each forward and reverse pre-amplification primer (Additional file 1: Table S2), and 7.5 units Platinum Taq polymerase (Invitrogen)], using the same amplification protocol as previously described [25]. Prior to qPCR, the products were diluted 5-fold in 1x Tris-EDTA buffer.

*Detection by real-time quantitative PCR –* Prior to qPCR an incubation step was performed to digest any leftover pre-amplification primers in the solution. The diluted DNA products were transferred to a PCR plate and mixed with 1.4x Fast Universal Master Mix (Applied Biosystems), dH_2_O and 0.05 units of uracil-DNA excision mix (Epicenter) and then incubated for 30 min at 37°C. The qPCR detection was performed on either an ABI 9700 HT Fast (Applied Biosystems) instrument or the BioMark^TM^ micro fluidic system (Fluidigm). Four μl of each pre-amplification product were transferred to a qPCR plate and mixed with 6 μl qPCR mix [25 mM Tris–HCl, 7.5 mM magnesium chloride, 50 mM potassium chloride, 8.3 mM ammonium sulfate, 8.3% Trehalose (Acros Organics), 333 μM (each) dNTP’s, 1.67 mM dithiothreitol, 833 nM of each primer (Additional file 1: Table S2), 417 nM Molecular Beacon (Biomers), 41.7 U/ml recombinant Taq polymerase (Fermentas) and 1.33 μM ROX reference (ROX-TTTTTTT, Biomers)]. The thermal cycler program was two-step with initial denaturation at 95°C for 5 min, followed by 15 s denaturation at 95°C; and 1 min annealing/extension at 60°C for 45 cycles.

#### Implementation procedure

*Pre-amplification –* was performed in a total volume of 25 μl by mixing 20 μl of the ligated product with 5 μL PCR mix [1x PCR buffer (Invitrogen, Denmark), 15 mM MgCl_2_ (Invitrogen), 1 mM dNTP (Invitrogen), 0.2 μM of each forward and reverse pre-amplification primer (Additional file 1: Table S2), and 7.5 units Platinum Taq polymerase (Invitrogen)], using the same amplification protocol as previously described [25]. Prior to qPCR, the products were diluted 5-fold in 1x Tris-EDTA buffer.

*Detection by real-time quantitative PCR –* Prior to qPCR an incubation step was performed to digest remaining pre-amplification primers in the solution. Ten μl of the 2-fold diluted DNA product was mixed with 10 μl 1.4x Fast Universal Master Mix (Applied Biosystems), dH_2_O including 0.05 units of uracil-DNA excision mix (Epicenter) and then incubated for 30 min at 37°C. Afterwards, each sample mix was further diluted by adding 80 μl Tris-EDTA buffer. The qPCR detection was performed on a 384-well format using the LightCycler 480. Four μl of each diluted pre-amplification product were transferred to a well in the 384-plate and mixed with 6 μl qPCR mix. Again, the thermal cycler program was 95°C for 5 min, followed by 15 s denaturation at 95°C; and 1 min annealing/extension at 60°C for 45 cycles.

#### Preparation of internal standards, standard curves, and samples for recovery studies

For internal control standard we used recombinant GFP (Vector Laboratories) which was spiked in the spike-in mix (Olink Bioscience) and thereby added to all sample incubations. GFP was diluted in PBS + 0.1% BSA (Calbiochem/Merck) to a final concentration of 10 nM.

When analyzing qPCR data it is important to consider the scale of the Ct values. Differences of 1 Ct is similar to a two-fold difference on a linear scale, and a higher Ct value indicates lower concentration. To convert the Ct-values to a linear scale, we used the formula 2^(*40-Ct)*^. These linearized data were then normalized to the internal control GFP by dividing each biomarker value of one sample with the value for GFP for this sample. This first normalization reduces the technical variation and improves the data quality significantly. To compensate for shifts in the different runs, we also normalized between each qPCR run (total of three chips). By assuming that such a large sample set will have the same expected median, we divided each assay with the median value for all samples on that chip. The result of this second normalization was that we reduce variability introduced between qPCR runs. Together with a buffer control a spike-in of recombinant protein mix (each 200 pM) PBS + 0.1% BSA was added to each qPCR plate.

#### Correlations

In order to evaluate the PEA, data were correlated with previously obtained ELISA data [25] utilizing three commercially available ELISAs for quantification (Carcinoembryonic antigen-related cell adhesion molecule 5 (CEA), Interleukin-8 (IL-8) and cancer antigen 242 (CA242)) and an in-house validated ELISA for TIMP-1 [26]. Results were obtained from the sample cohort of healthy individuals and CRC patients. Furthermore, PLA data from a previous run [25] were used to evaluate the PEA assay performance. Pearson correlation between PLA and PEA data was calculated and the Pearson correlation value (R) presented. Logarithmic PLA values were linearized 2^(35-Ct)^ and normalized with GFP prior to comparison to the ELISA values. Logarithmic PEA values were normalized against both the median for each assay and GFP and linearized 2^(35-Ct)^ prior to comparison to ELISA values.

#### Statistical analysis

Candidate markers for analysis were selected from the available molecular markers choosing those with p-values less than 0.001 (Type III) when comparing the patient groups using a linear model with repeated measures (cases). The chosen markers were then analyzed by logistic regression analysis adjusting for the case–control design with CRC versus adenomas, non-neoplasticdisease and healthy individuals. The probability of CRC was modeled. Each chosen marker was entered by the actual normalized value. Univariate analysis was performed for each marker and a multivariate model was identified retaining markers which were statistically significant. The results are presented by the odds ratio (OR) with 95% confidence interval (CI). Sensitivity and specificity were calculated for each marker and the linear combination was used for the multivariate model. In addition, ROC curves were generated and the area under the curve (AUC) was calculated. Subset analyses of CRC restricted to TNM stages I and II as well as comparison of adenomas to non-neoplastic and healthy subjects were performed. Cross-validation methods were used to assess the chosen models. P-values less than 5% were considered significant. All calculations were performed using SAS (v9.2, SAS Institute, Cary, N.C., USA).

#### Functional analysis in silico

Independence among biomarkers has shown to increase the additive effects of a panel of biomarkers [16-18]. In order to investigate the biological association between the top hit of our interesting biomarkers, we initiated a focused bioinformatics evaluation. Functional analysis of the four proteins of interest was performed by using IPA (Ingenuity® Systems, http://www.ingenuity.com). The protein identifiers for CEA, Transferrin Receptor protein 1 (TFRC), Macrophage migration inhibitory factor (MIF) and Osteopontin (OPN/SPP1) were uploaded to the application. The functional analysis identified the biological functions and diseases that were most significant to the four genes in the Ingenuity Knowledge Base. Right-tailed Fisher’s Exact Test was used to calculate a p-value determining the probability that each biological function and disease assigned to that data set is due to chance alone.

#### Assay exportation, implementation and validation

We exported the PEA technology from Olink Bioscience, Uppsala, Sweden to the University of Copenhagen, Copenhagen, Denmark and performed validation of the candidate biomarker assays. Sensitivity, specificity and linearity of signal were assessed by spike-in of recombinant protein in PBS + 0.1% BSA buffer in a range of 0.01 – 10,000 pM and standard curves. The specificity of each assay was assessed in PBS + 0.1% BSA by using different antigen mixes with or without the specific antigen present. Standard curves for three different specific mixes and one unspecific mix were assessed for each assay. The overview and composition of the different mixes are illustrated in (Additional file 1: Table S4). Contamination of the TIMP-1 PEA probe led us to exclude this assay from the validation process. No suitable commercially available CA242 and Cancer antigen 19–9 (CA19-9) recombinant proteins were available and therefore linearity in buffer and recovery could not be assessed for these assays. Intra- and inter-assay variations were calculated for the GFP control by calculating the standard deviation between GFP measurements within one plate and among plates (assays performed at University of Copenhagen). Linearity in CRC plasma was investigated for all assays by performing a 10-fold dilution in the range of undiluted to 10,000 times dilution.

Recovery studies were made in PBS + 0.1% BSA (expected) and human control plasma (measured) and the biological background cross-reactivity was assessed in chicken plasma (GeneTex, Cat.no. GTX73211). Recombinant proteins were spiked in PBS + 0.1% BSA or human control plasma in the range of 10, 100 and 500 pM. To assess the recovery, the Cp-values were linearized by the formula 2^*(40-Cp)*^ and then assessed by calculating the background = buffer + buffer with spike-in; the expected value = control plasma + background giving $\mathrm{recovery}\left(\%\right)=\frac{\mathrm{measured}}{\mathrm{expected}}\times 100$. The recovery was evaluated for nine assays for which recombinant proteins were available; the recovery was calculated for all spike-in concentrations and a range of these were then presented as the recovery.

## Results

### Assay optimization from PLA to PEA

To improve the assay performance and to overcome plasma inhibition of the ligase in the PLA protocol, the linkage between the two proximity probes was changed to an extension after hybridization of an extension oligonucleotide (PEA) [19]. We examined how PEA compared to previous ELISA data and, overall, the PEA data showed improved correlation to the ELISA data in comparison to the correlation between PLA and ELISA (performed in a previous study [25]). These results were calculated on the basis of ELISA, PLA and PEA measurements of the level of the specific markers in plasma from healthy individuals (n = 70) and CRC patients (n = 70). This was performed in order to complete an assessment of the newer techniques in comparison to the gold-standard, ELISA (Additional file 1: Table S5). Investigation of the PEA performance in comparison to the PLA was performed as χ2 statistic for the PLA and PEA covariates from the multivariate logistic regression analysis for each biomarker. The χ2 statistic shows a larger value for the PEA in all cases demonstrating that the PEA covariate is the best discriminator of CRC. In order to examine the relationship between PLA and PEA we performed a Pearson correlation between each biomarker entering either PLA or PEA. The Pearson correlation coefficients demonstrated a substantial association between PLA and PEA levels (Additional file 1: Table S6). Univariate analysis for each biomarker entering either the PLA or the PEA value also shows that the PEA covariate yields the best model fit (data not shown) for each tested biomarker.

### Statistical evaluation of potential colorectal neoplasm biomarkers

**Figure 1:**
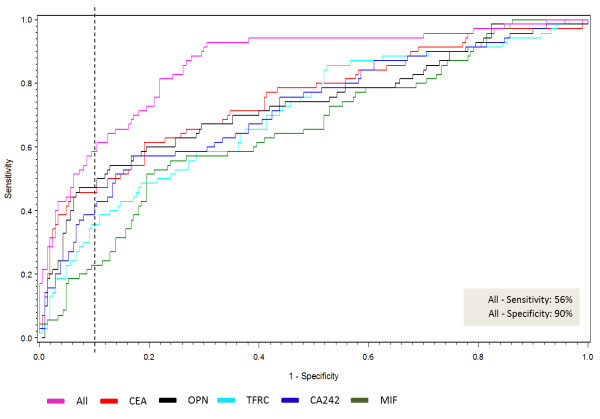
**Receiver operating characteristic (ROC) curve modeling the candidate CRC biomarkers’ (CEA, TFRC, MIF, OPN/SPP1 and CA242) probability of colorectal cancer detection.** Data included are from colorectal cancers versus controls for individual biomarkers (n = 140). The x axis is 1-specificity and the y axis is sensitivity. The purple line represents the combination of all five markers with maximum sensitivity and specificity of 56% and 90%, respectively. The area under the ROC curve (AUC) signifies the accuracy of each of the individual as well as the combined biomarkers for distinguishing colorectal cancers. The AUCs ranged from 0.658-0.731. The dotted black line represents the point of 90% specificity and relationship is indicated by color.

A total of 74 different biomarkers were analyzed in the 4 x 70 human plasma samples (Additional file 1: Table S1). Twelve biomarkers discriminated between CRC patients and healthy individuals in a univariate analysis (P < 0.001) (Table 2). These 12 protein biomarkers were included in a multivariate analysis to evaluate their statistical association. This analysis demonstrated that 5 of the 12 different proteins significantly identified CRC from the control groups; CEA (P = 0.0003), TFRC (P = 0.0007), MIF (P = 0.0068), OPN/SPP1 (P = 0.0200) and CA242 (P = 0.0090) (Table 2). The AUC for the individual proteins ranged from 0.658 to 0.731 and sensitivities were: CEA 46%, TFRC 34%, MIF 38%, OPN/SPP1 38% and CA242 39% at 90% specificity (Figure 1). Estimating ROC curves from this conditional logistic regression analysis illustrated that the five CRC discriminators (CEA, TFRC, MIF, OPN/SPP1 and CA242) had an additive effect, as their combined curve demonstrated an increase in AUC with a sensitivity of 56% at 90% specificity. Additional, we investigated the Pearson correlation coefficients between the 12 potential CRC biomarkers, which were selected on a basis of 0.01% discrimination, in order to investigate the association among the markers. The association intervals (R’s) for the five potential biomarkers were; TFRC (0.05 – 0.40), MIF (0.05 – 0.81), CEA (0.19 – 0.41), CA242 (0.07 – 0.61) and OPN (0.07 – 0.50). The association among the five markers was in the lower range. Markers with a strong correlation would likely not be independent in multivariate analysis (Additional file 1: Table S7).

**Table 2:** Uni- and multivariate statistical analyses of the 12 potential screening markers selected on the basis of a 0.01% discrimination

**Univariate analysis**					**Multivariate analysis**			
**TNMI-IV (N = 70 versus 210)**			**Adenoma (N = 70 versus 140)**		**TNMI-IV**^**a **^**(N = 70 versus 210)**		**TNMI-II**^**b **^**(N = 36 versus 108)**	
	**Odds ratio (95% CI)**	**P**	**Odds ratio (95% CI)**	**P**	**Odds ratio (95% CI)**	*P	**Odds ratio (95% CI)**	**P**
CEA	2.2 (1.7-2.9)	<0.001	1.2 (0.9-1.7)	0.2600	1.8 (1.3-2.4)	0.0003	2.0 (1.3-3.0)	0.0007
TFRC	2.8 (1.9-4.2)	<0.001	1.0 (0.7-1.5)	0.9600	2.7 (1.5-4.8)	0.0007	2.1 (1.1-4.1)	0.0303
CA242	2.2 (1.7-3.0)	<0.001	1.0 (0.7-1.3)	0.7700	1.6 (1.1-2.3)	0.0090	1.8 (1.1-3.0)	0.0311
OPN/ SPP1	13.4 (4.6-39.0)	<0.001	1.7 (0.7-4.2)	0.2200	5.5 (1.3-23.1)	0.0200		
MIF	3.0 (1.8-5.1)	<0.001	1.6 (1.0-2.5)	0.0400	2.6 (1.3-5.1)	0.0068		
NSE	2.0 (1.4-2.8)	0,0001	1.5 (1.1-2.2)	0.0240		0.6000		
CA19-9	1.5 (1.2-1.8)	<0.001	1.1 (0.9-1.3)	0.4500		0.5900		
DcR3	1.7 (1.3-2.7)	0.0005	1.3 (1.0-1.8)	0.1100		0.6500		
IL8	2.4 (1.7-3.5)	<0.001	1.2 (0.9-1.7)	0.2700		0.1500		
S100A8	4.9 (2.3-10.4)	<0.001	1.4 (0.7-2.7)	0.3300		0.8600		
TIMP1	2.4 (1.6-3.5)	<0.001	1.3 (0.9-1.9)	0.1300		0.1700		
TFF3	2.2 (1.5-3.1)	<0.001	1.0 (0.7-1.3)	0.8100		0.3500		

*P-value to include in final model. ^a^ Including all CRC stages (TNMI-VI) in a conditional logistic regression model; ^b^ Including only the early CRC stages (TNMI-II) in a conditional logistic regression model.

Univariate analyses of the markers as discriminators of TNM stage I-IV demonstrate that all markers can discriminate CRC (n = 70) from the three control groups (n = 210). When including these markers in a multivariate analysis we find that five markers are still discriminators of CRC (n = 70). Including only CRC TNM I-II patients in the multivariate analysis we find that three markers are continuously discriminators of CRC. Univariate analyses of the markers as discriminators of adenomatous disease demonstrate that only one marker (NSE) is discriminator of adenoma (n = 70) and the two control groups, patients with other diseases and healthy individuals (n = 140).

The adenoma patients were included in the study since surgical excising of adenomas has previously demonstrated a decrease in the incidence of CRC and thereby an increase in survival of screened individuals compared to non-screened individuals [27]. Except for NSE (P = 0.0240) and MIF (P = 0.040), none of the markers were able to discriminate between the control groups (healthy individuals and patients with other diseases) and the adenoma patients in the univariate analysis (Table 2). Furthermore, for the adenoma group NSE was the only marker which was not associated with the other markers and hence we could not investigate any additive effect of potential adenoma-specific markers.

### Statistical evaluation of potential early-stage CRC biomarkers

**Figure 2:**
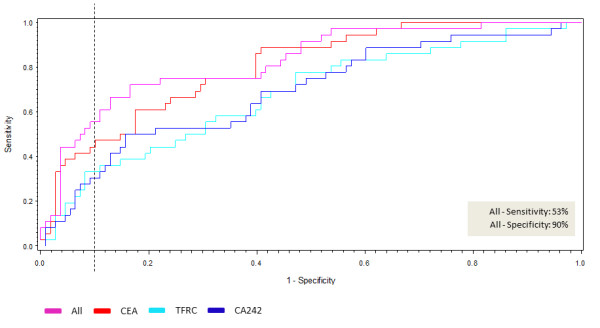
**Receiver operating characteristic (ROC) curve modeling the probability of colorectal cancer detection by CEA, TFRC and CA242.** Data from colorectal cancer stage TNM I-II versus matched controls were included in the statistical calculation (n = 72). The x axis is 1-specificity and the y axis is sensitivity. The red line represents the combination of all three markers with maximum sensitivity and specificity of 53% and 90%, respectively. The area under the ROC curve (AUC) signifies the accuracy of the three combined biomarkers for distinguishing colorectal cancers (TNM I-II). The area under the curve was 0.861. The dotted black line represents the point of 90% specificity.

The alterations of cancer biomarkers are often correlated to increase in tumor size and stage [13,28]. Hence, we investigated the interesting markers when including only early-stage CRC patients. We applied our discrimination model to early-stage CRC by only including CRC TNM I-II patient and biomarker data. This demonstrated that three of the five CRC biomarkers could identify early-stage CRC (n = 36) from the controls (n = 36) in each control set; CEA (P = 0.0007), TFRC (P = 0.0303) and CA242 (P = 0.0311) (Table 2). Their combined performance was evaluated by plotting a ROC curve of the output of these three identifiers. The AUC is 0.69 for CA242, 0.68 for TRF and 0.80 for CEA, whereas the combined ROC curve demonstrated that the combination of the three biomarkers resulted in an AUC of 0.82 (Figure 2). Combinations of the three biomarkers showed 53% sensitivity at 90% specificity.

### Functional analyses in silico

**Figure 3:**
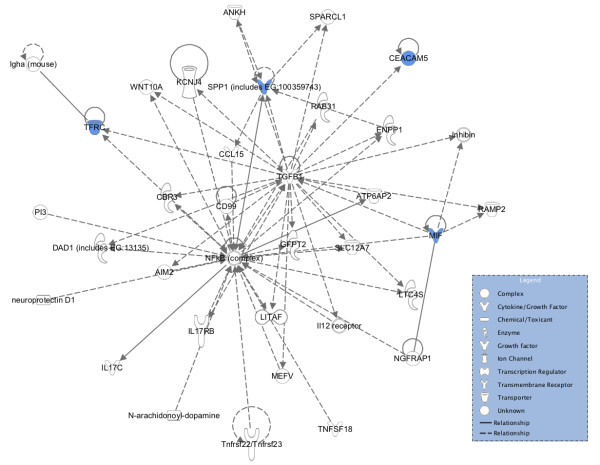
**Graphical representation of the molecular relationships among the candidate CRC biomarkers identified in human plasma; CEA, TFRC, MIF and OPN/SPP1.** The top associated network was the Organ Morphology, Cardiovascular Disease, Cellular Development network. Molecule types have their background highlighted as described in the blue box. Edges with dashed lines show indirect interaction, while an unbroken line represents direct interactions. Molecules in uncolored notes were integrated into the computationally generated networks on the basis of the evidence stored in the IPA knowledge memory indicating a relevance to this network.

Functional analyses identified the biological functions and diseases that the four biomarkers, CEA, TFRC, MIF and OPN/SPP1, were most significantly associated with. CA242 was not included in the analysis since it is defined as a blood-group antigen [29] and it was not registered in the IPA library (Ingenuity® Systems, http://www.ingenuity.com). By performing canonical pathways analysis we identified the pathways from the IPA library that were most significant to the four biomarkers: Eumelanin Biosynthesis (MIF), MIF-mediated Glucocorticoid Regulation (MIF), MIF Regulation of Innate Immunity (MIF), Role of Oct4 in Mammalian Embryonic Stem Cell Pluripotency (OPN/SPP1) and VDR/RXR Activation (OPN/SPP1). The pathway analysis demonstrated no common pathways between any of the four biomarkers. The functional network analysis demonstrated that all four biomarkers were found in the network “Organ Morphology, Cardiovascular Disease, Cellular Development” which is illustrated in Figure 3.

### Assay exportation, implementation and validation

**Figure 4:**
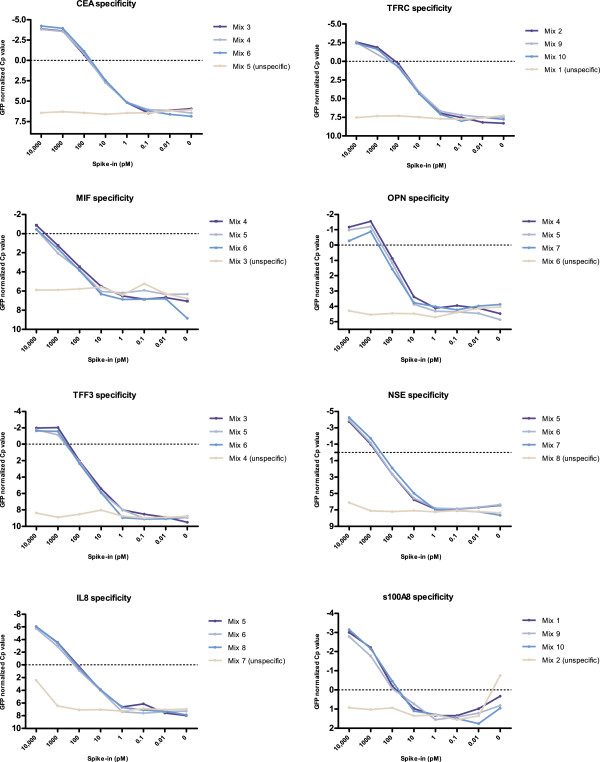
**Dose–response and technical specificity for candidate biomarkers.** Technical specificity is assessed by comparing standard curves prepared from a serial dilution of antigen mixes with (specific) or without (unspecific) the specific antigen present. These different solution mixes are PBS + 0.1% BSA ± antigen (Additional file 1: Table S4). The x-axis shows the antigen concentrations in the different mixes. The y-axis shows Cp-values, which were normalized to the internal control GFP by subtracting each biomarker value from the GFP-value for this sample. As indicated, each assay was tested by three different specific antigen mixes and a single unspecific antigen mix, which indicate the background of the assay.

The portability of PEA among independent laboratories was obtained successfully. Linearity of signal, sensitivity and specificity were investigated for eight assays in buffer with spike-in recombinant protein and demonstrated linear ranges of 3–4 orders of magnitude, sensitivity in a range of 1–10 pM and high specificity for all assays, except for the s100A8 assay, which demonstrated a high background level (Figure 4). All assays had a low background level in chicken plasma, demonstrating that no unspecific binding as well as no stickiness to the biological components of chicken plasma was present for the antibodies and oligonucleotides of the PEAs (Additional file 1: Figure S1). The CV% was calculated for GFP by linearizing the logarithmic raw Cp values and then calculating the average and standard deviation. For intra-assay variation determinations, 15 different experiments were performed with eight measurements in each. The CV% ranged from 9.0-31.5; median CV% = 16.9 and average CV% = 17.6. The inter-assay variation was calculated among the 15 experiments and the CV% was found to be 34.3 (n = 144). Linearity in plasma was assessed in order to evaluate if the measurement of each biomarker was within the linear range of the specific assay. All assays with proximity probes available demonstrated an acceptable linear range in human plasma (Additional file 1: Figure S2). However, the linear ranges of s100A8 and TGFb1 show high background level and hence a lower linear range. The ideal plasma dilution for the CRC sample was shown to be a factor of 10, as this was within the linear range of the 11 biomarker assays tested. Unfortunately, the TIMP-1 assay was excluded from these experiments due to a contamination of the TIMP-1 probes. Recovery was assessed in human control plasma for the nine assays with antigens available for the experiment, the recovery ranged from 58-129% between assays (Additional file 1: Table S3).

## Discussion

The present study demonstrates that the novel multiplex PEA assay is suitable to identify potential plasma biomarkers for detection of CRC. Forming a biomarker panel consisting of plasma CEA, TFRC, MIF, OPN/SPP1 and CA242 could represent a biomarker test for detection of CRC in symptomatic individuals. Of particular interest is the observation that the plasma levels of CEA, TFRC and CA242 were identifiers of early-stage CRC, suggesting that the panel holds potential as an early detection method of CRC. However, future studies including asymptomatic individuals as well as patients with other diseases, including other cancer types, are needed to test this hypothesis. We also investigated plasma obtained from adenoma patients in order to search for a precancerous biomarker, but unfortunately no adenoma-specific biomarkers with sufficient statistical significance could be identified.

Among symptomatic individuals the five CRC biomarkers identified were shown to detect CRC patients with a sensitivity of 56% at a specificity of 90% (Figure 1). Furthermore, functional in silico analyses showed that the four biomarker proteins are involved in different pathways, which supports their lack of association and their individual contribution to the additive effect of the panel. These four biomarkers did not have any direct interactions, yet all had interactions with TGFβ1, indicating some common functional character. The most significant associated diseases and disorders associated with the four biomarkers were inflammatory response (CEA, MIF, OPN/SPP1), cancer (all four), gastrointestinal disease (all four), cardiovascular disease (MIF, OPN/SPP1, TFRC) and infectious disease (MIF, OPN/SPP1, TFRC), supporting that these biomarkers have a biological role in CRC. The three early-stage CRC biomarkers were shown to identify stage I and II CRC patients with a sensitivity of 53% at 90% specificity (Figure 2). In asymptomatic individuals, the sensitivities of the FOBT range from 40-90% at specificities ranging from 85-90% [8], but the sensitivity is highly affected by the low compliance for faeces tests [7,30]. Nevertheless, it has been shown that CRC screening by the FOBT reduces mortality from CRC by 15-21% [6,31,32] and that CRC screening in general increases the number of patients detected with early-stage disease [33]. Measurements of SEPT9 methylated DNA in serum of asymptomatic individuals have recently been shown to identify CRC patients with a sensitivity of 67% at 89% specificity [11]. The performance of our biomarker panel is thus within the ranges of the FOBT and almost within the ranges of the SEPT9 test, although a direct comparison between asymptomatic and symptomatic individuals may be inaccurate. Furthermore, like the SEPT9 test, our biomarker panel has the advantage of being a simple blood test and thus the compliance will most probably be high. It should be noted, however, that our results are obtained from analysis of symptomatic patients prior to endoscopy. Initiating a validation study in an asymptomatic population-based cohort might result in altered sensitivity and specificity performance as well as an altered compliance.

To obtain further information about the value of our biomarker panel as identifier of early-stage CRC we compared the analysis of CRC TNM stage I-II with their matching control groups (healthy individuals, other diseases and adenoma patients). We found three positive identifiers; TFRC, CEA and CA242 with known biological roles and previous associations to cancer. TFRC has an important role in the inflammation process and it has also been described as being specific for tumor cell proliferation as it provides high iron uptake required for cell division [34,35]. CEA is a glycoprotein and a well-known CRC monitoring marker [19,36] suggested to mediate cell-cell adhesion, maintain the bacterial environment of the intestine and protect the colon from infectious microorganisms [36]. Lastly, CA242 is a blood-group antigen defined by the monoclonal antibody C242 and it has also previously been described as a potential CRC biomarker [37]. The adenoma patients were included in the study as it would be an advantage to include adenoma biomarkers in a potential screening panel. However, only NSE was a significant identifier of adenoma, but it did not demonstrate any impact in the ROC analysis. The low success rate of adenoma biomarkers could be a consequence of our initial literature search from which the 74 proteins were initially chosen as the search was focused on CRC biomarkers.

A challenge of both adenoma and early-cancer serological markers is the concentration of each analyte which needs to be of a sufficient level in the blood before it is theoretically possible to measure it [38]. From the literature we know that CEA is increased in tumor tissue [39], that the *MIF* (Hs.407995) is up-regulated in colorectal carcinomas [40], that OPN is significantly higher in CRC tumor tissue compared to normal tissue [41] and that TFRC is higher in tumor tissue compared to normal mucosa [42]. The tumor tissue level of CA242 is not well established. Searching for detection markers in the early CRC stages when the tumor is minor or in the adenomateous lesions it has been argued that the production of protein markers is not sufficient to make an imprint in the systemic circulation [42]. This could be a potential pitfall for discovery of biomarkers, especially for the early stage CRC and the adenomateous lesions, as one would expect less activity from these. However, to take one example, the TIMP-1 plasma level has been described by several authors to be elevated in CRC patients. In most of these studies it was found that stage I, II and III CRC patients had the same degree of TIMP-1 elevation as compared to non-neoplastic individuals and thus the plasma level was not related to tumor burden [43]. In the present study we were able to statistically point out discriminators of both CRC stage I-II and all stage CRC in plasma suggesting that it is possible to develop serological screening markers for CRC. The origin of these markers is unknown and could be derived from the tumor microenvironment rather than the tumor itself. This question needs yet to be answered and it would be interesting to compare the tumor tissue level with the serological level of each of the identified CRC biomarkers.

## Conclusions

Survival after CRC is currently being improved by screening, but improvement of detection methods for early stage CRC is needed. Such methods should be easy to perform, have high sensitivity, high specificity and high compliance and be inexpensive. Since CRC is a heterogeneous disease it could be an option, as indicated in the present study, to combine different screening tests in order to obtain a high test performance. Consequently, implementation of a sensitive and specific serological screening test could be of great importance as an add-on to the FOBT or SEPT9. In that regard it could be an advantage that the test platforms are run on the same specimen in order to simplify sample collection and maintain high compliance. Interestingly, proximity assays may be applied for analysis of stool samples [44] and saliva (unpublished observations) which could add further to the usability of this assay in CRC biomarker research.

In summary, five plasma protein biomarkers were identified by the novel multiplex PEA assay as potential CRC discriminators and three of these were additionally found to be discriminators of early-stage CRC. The performance of our biomarker panel in symptomatic individuals was within the range of sensitivity and specificity seen for asymptomatic individuals applying the FOBT and the performance of the SEPT9 test. PEA assays of the five identified protein biomarkers have been thoroughly validated. However, analyses in a new independent and larger sample cohort of asymptomatic individuals are needed in order to further validate the performances of our CRC biomarker panel.

## Abbreviations

CA19-9: Cancer antigen 19–9; CA242: Cancer antigen 242; Ab: Antibody; Ag: Antigen; AUC: Area under the curve; CEA: Carcinoembryonic antigen; CI: Confidence interval; Ct: Cycle threshold; CV: Coefficient of variation; FOBT: Faecal occult blood test; GFP: Green fluorescent protein; IL-8: Interleukin-8; mAb: Monoclonal antibody; MIF: Macrophage migration inhibitory factor; nt: Nucleotides; OPN/SPP1: Osteopontin; OR: Odds ratio; pAb: Polyclonal antibody; PEA: Proximity extension assay; PLA: Proximity ligation assay; ROC: Receiver operating characteristic; RT: Room temperature; SEPT9: Septin 9; sDNA: Stool DNA; SOP: Standard operating procedure; TFRC: Transferrin Receptor-1.

## Competing interests

M.L., E.A., A.V. and S.F. are employees at Olink Bioscience AB owner of intellectual property on the proximity ligation assay.

## Authors’ contributions

EA, JS, SF, AV, HJN and NB conceived the study and coordinated activities. HJN collected all samples and information on the patients. ML, SBT, SLCT, BSN, SF and EA established, optimized, validated and performed the proximity assays. MK and NG optimized the conjugation step in the probe preparation process. IJC performed all statistical analyses. SBT, NB, and JS drafted the manuscript. All authors read and approved the final manuscript.

## Authors’ information

Stine Buch Thorsen and Martin Lundberg: Shared authorship.

Jan Stenvang and Erika Assarsson Shared senior authorship.

## Supplementary Material

Additional file 1: Figure S1 Specificity test in chicken plasma. **Figure S2**. Linearity in human CRC plasma (n = 1). **Table S1**. All 74 biomarkers and controls divided into panel. **Table S2**. Oligonucleotide and qPCR primer sequence design. List of 3′ and 5′ oligonucleotide sequences used for antibody conjugations forming PEA probes along with hybridization oligonucleotide, extension oligo and primer sequences for pre-amplification and quantitative real-time PCR. **Table S3**. Recovery (%) for nine of the PEA assays after technology transfer. **Table S4**. Antigen mixes. **Table S5**. Correlation Coefficients between ELISA/PLA and ELISA/PEA. **Table S6**. PLA- and PEA correlations. **Table S7**. Intra-variation. Click here for file
