# Supplementary material for: Detection of serological biomarkers by proximity extension assay for detection of colorectal neoplasias in symptomatic individuals
Source: J Transl Med. 2013 Oct 10;11:253. doi: 10.1186/1479-5876-11-253 (PMC3827929; doi:10.1186/1479-5876-11-253)
Supplement: Additional file 1: Figure S1 — Specificity test in chicken plasma. Figure S2. Linearity in human CRC plasma (n = 1). Table S1. All 74 biomarkers and controls divided into panel. Table S2. Oligonucleotide and qPCR primer sequence design. List of 3′ and 5′ oligonucleotide sequences used for antibody conjugations forming PEA probes along with hybridization oligonucleotide, extension oligo and primer sequences for pre-amplification and quantitative real-time PCR. Table S3. Recovery (%) for nine of the PEA assays after technology transfer. Table S4. Antigen mixes. Table S5. Correlation Coefficients between ELISA/PLA and ELISA/PEA. Table S6. PLA- and PEA correlations. Table S7. Intra-variation. [file 1479-5876-11-253-S1.pdf]

Detection of serological biomarkers by Proximity Extension Assay for detection of colorectal neoplasias in symptomatic individuals

Stine Buch Thorsen[1,a], Martin Lundberg[2,a], Andrea Villablanca[2], Sarah Tangsgaard[1], Kirstine Christensen Belling[3], Birgitte Sander Nielsen[1], Mick Knowles[4], Nick Gee[4], Hans Jørgen Nielsen[5], Nils Brünner[1], Ib Jarle Christensen[6], Simon Fredriksson[2], Jan Stenvang[1,b,*] and Erika Assarsson[2,b]

[a]Shared authorship

[b]Shared senior authorship

[1]Department of Veterinary Disease Biology, Faculty of Health and Medical Sciences, University of Copenhagen, Strandboulevarden 49, DK-2100, Copenhagen, Denmark

[2]Olink Bioscience, Uppsala Science Park, Dag Hammarskjölds väg 52B, SE-75237 Uppsala, Sweden

[3]Department of Systems Biology Center for Biological Sequence Analysis, Technical University of Denmark DTU, DK-2800 Kgs. Lyngby, Denmark

[4]Innova Biosciences, Babraham Hall, Cambridge, CB22 3AT, United Kingdom

[5]Department of Surgical Gastroenterology, Hvidovre Hospital, DK-2650 Hvidovre, Denmark

[6]The Finsen Laboratory, Rigshospitalet, DK-2200 Copenhagen N, Denmark and Biotech Research and Innovation Center (BRIC), University of Copenhagen, DK-2200 Copenhagen N, Denmark

*To whom correspondence should be addressed: Jan Stenvang, Tel: +45 35333753; Fax : +45 35332755; Email: stenvang@sund.ku.dk

Additional Materials

addFigure 1 – Specificity test in chicken plasma

addFigure 2 – Linearity in human CRC plasma (n=1)

addTable 1 – All 74 biomarkers and controls divided into panels

addTable 2 – Oligonucleotide and qPCR primer sequence design. List of 3' and 5' oligonucleotide sequences used for antibody conjugations forming PEA probes along with hybridization oligonucleotide, extension oligo and primer sequences for pre-amplification and quantitative real-time PCR.

addTable 3. Recovery (%) for nine of the PEA assays after technology transfer. Recovery is presented for all assays of which a commercial antigen was available.

addTable 4 – Antigen mixes.

addTable 5 – Correlation Coefficients between ELISA/PLA and ELISA/PEA

addTable 6. PLA- and PEA correlations

addTable 7 – Intra-variation

addFigure 1 – Specificity test in chicken plasma. The signal in buffer (PBS + 0.1% BSA) in comparison to the signal in chicken plasma. The y-axis shows Cp-values and no differences were observed among the signals for the individual assays when buffer and chicken plasma was compared. This indicates that the assays possess very low biological background cross-reactivity and demonstrates no unspecific binding to the biological components of chicken plasma for the antibodies and oligonucleotides of the PEA assays.

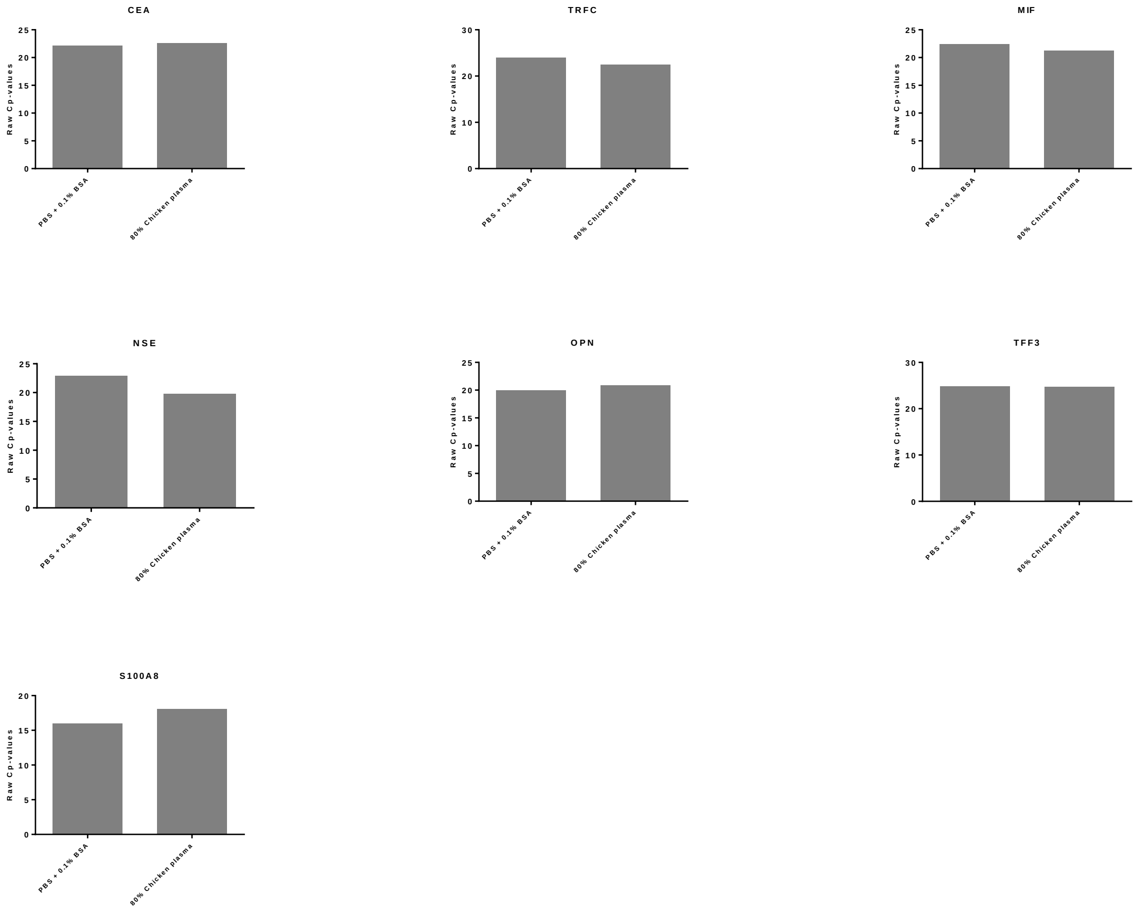

addFigure 2 – Linearity in human CRC plasma (n=1). The x-axis shows the dilution factor of the sample. The y-axis shows the Cp-values, which were normalized to the internal control GFP by subtracting each biomarker value from the corresponding GFP-value for each sample. The ideal plasma dilution for the CRC sample is 10, which is within the linear range of the present 11 potential markers. TIMP-1 was excluded due to a technical issue.

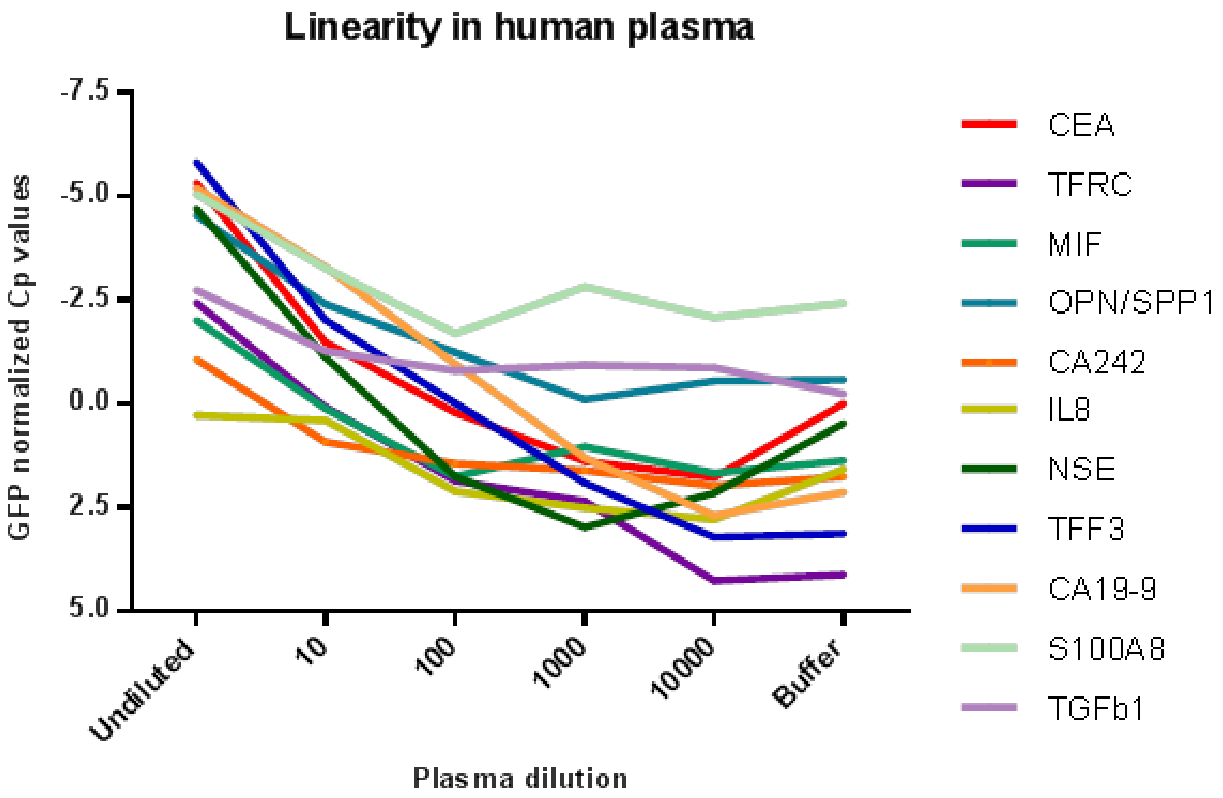

addTable 1

Seq No	Panel 1 Marker	Antibody Supplier	Catalog ID#1/ Catalog ID#2	Uniprot ID
1	Carbonic Anhydrase IX	RnD systems	AF2188	Q16790
2	MCP-1 (CCL2)	RnD systems	AF279-NA	P13500
3	Mesothelin	RnD systems	AF3265	Q13421
4	IL-6	RnD Systems	AF-206-NA	P05231
5	No assay	RnD Systems	AF2286	-
6	FAM3D	RnD systems	AF2869	Q96BQ1
7	CEACAM 5 pAb	RnD systems	AF4128	P06731
8	VEGF	RnD systems	AF-293-NA	P15692
9	No assay			-
10	No assay			-
11	CEA mAb	USBio	C1300-02/C1300-07	P06731
12	CA125	FDAB	22582 ov197/22781 ov185	-
13	Her4/erbB-4	RnD systems	AF1131	Q15303
14	HGF/SF	RnD systems	AF-294-NA	P08581
15	FABP2	RnD systems	AF3078	P12104
16	Oligo PCR	-	-	-
17	APC	Novus	NB100-78112	-
32	GFP	RnD systems	AF4240	-
35	IL8	RnD systems	AF-208-NA	P10145
38	PE	Abcam		-
45	Her2/ERBB2	RnD systems	AF1129	P04626
51	Her3	RnD systems	AF234	P21860
53	TNFa	RnD systems	AF-210-NA	P01375
57	IL-1a	RnD systems	AF-200-NA	P01583

Seq No	Panel 2 Marker	Antibody Supplier	Catalog ID#1/ Catalog ID#2	Uniprot ID
1	Survivin	RnD systems	AF6471	Q15392
2	Desmin	RnD systems	AF3844	P17661
3	HGF R/cMET	RnD systems	AF276	P08581
4	IL-17	RnD systems	AF177	Q9NRM6
5	IL-23	RnD systems	AF1716	Q9NPF7
6	FoxP3	RnD Systems	AF3240	Q9B251
7	CEACAM 5 pAb	RnD systems	AF4128	P06731
8	CEACAM1	RnD systems	AF2244	P13688
9	TGFb1	RnD systems	AF-246-NA	P01137
10	TGFb3	RnD systems	AF-243-NA	P10600
11	KLK3 (PSA)	RnD systems	AF1344	P07288
12	Cystatin SN	RnD systems	AF1285	P01037
13	Prolactin	RnD systems	AF682	P01236
14	DcR3	RnD systems	AF142	O95407
15	IL8	RnD systems	AF-208-NA	P10145
16	Oligo PCR	-	-	-
17	APC	Novus	NB100-78112	-
32	GFP	RnD systems	AF4240	-
35	CTGF	RnD systems	AF660	P29279
38	PE	Abcam		-
45	GDNF	RnD systems	AF-212-NA	P39905
51	Fractalkine (CX3CL1)	RnD systems	AF365	P78423
53	MIP-1	RnD systems	AF-270-NA	P10147
57	CA 242	FDAB	C241 B24645/C242 B24650	NA

Seq No	Panel 3 Marker	Antibody Supplier	Catalog ID#1/ Catalog ID#2	Uniprot ID
1	Spondin-2/Midin	RnD systems	AF2188	Q9BUD6
2	EPCAM	RnD systems	AF960	P16422
3	NSE (enolase 2)	RnD systems	AF5169	P09104
4	Transaldolase 1	Sigma Aldrich	SAB1400273	P37837
5	No assay			-
6	Galectin-3	RnD systems	AF1154	P17931
7	TIMP1	RnD systems	AF970	P01033
8	EGFR	RnD systems	AF231	P00533
9	TFF3	RnD systems	AF4407	Q07654
10	u-PA	RnD systems	AF1310	P00749
11	CEA mAb	Stratech (UK)	http://www.stratech.co.uk/ shopexd.asp?id=144884&bc=no	P06731
12	Transferrin Receptor (CD71)	RnD systems	AF2474	P02786
13	CA 15.3	FDAB	22480 Ma552/22637 Ma695	NA
14	CA19-9	FDAB	c192 Mab 103-01	NA
15	Tropomyosin	http://antibodies.sdix.com	2272.00.02	P06753
16	Oligo PCR	-	-	-
17	APC	Novus	NB100-78112	-
32	GFP	RnD systems	AF4240	-
35	OPN	RnD systems	AF1433	P10451
38	PE	Abcam		-
45	MIF	RnD systems	AF-289-PB	P14174
51	No assay			-
53	SLPI	RnD systems	AF1274	P03973
57	S100A8	RnD systems	AF4570	P05109

Seq No	Panel 4 Marker	Antibody Supplier	Catalog ID#1/ Catalog ID#2	Uniprot ID
1	Clusterin	RnD systems	AF2188	P10909
2	No assay			-
3	sP-selectin	RnD systems	AF137	Q14242
4	MMP-2	RnD Systems	AF902	P08253
5	MMP-9	RnD Systems	AF911	P14780
6	Tenascin-C	RnD systems	AF3358	P24821
7	TIMP1	RnD systems	AF970	P01033
8	CRP	RnD systems	AF1707	P02741
9	PAI-1 / Serpin	RnD systems	AF1786	P05121
10	sVCAM/CD106	RnD systems	AF809	P19320
11	IGF-I	RnD systems	AF291	P08069
12	Cathepsin B	RnD systems	AF953	P07858
13	Angiogenin	RnD systems	AF265	P03950
14	IGF2	RnD systems	AF2447	P11717
15	Tetranectin	RnD systems	AF5170	P05452
16	Oligo PCR	-	-	-
17	APC	Novus	NB100-78112	-
32	GFP	RnD systems	AF4240	-
35	Cystatin C	RnD systems	AF1196	P01034
38	PE	Abcam		-
45	IGFBP-1	RnD Systems	AF871	P08833
51	GRP78/HSPA5/BiP	RnD Systems	AF4846	P11021
53	Kallikrein 11	RnD systems	AF1595	Q9UBX7
57	YKL-40	RnD systems	AF2599	P36222

addTable 2

Oligonucleotide design		
**Mux sequence**	**Sequence as 3' oligo**	**Sequence as 5' oligo**
1	CAGGTAGTAGTACGTCTGTTTCACGATGAGACTGGATGAA	TCACGGTAGCATAAGGTGCAAGATAATACTCTCGCAGCAC
2	GGCCTCCTCCAATTAAAGAATCACGATGAGACTGGATGAA	TCACGGTAGCATAAGGTGCAGTACCCAAATAACGGTTCAC
3	GGATCACTCCAACTAGACTATCACGATGAGACTGGATGAA	TCACGGTAGCATAAGGTGCAGTTATATCTGCCACTGTCAC
4	AGAGTCCACTTCCCATAATGTCACGATGAGACTGGATGAA	TCACGGTAGCATAAGGTGCACGGTACTGTCAGCATAGTTC
5	CTACGACTAGGAGATAGATGTCACGATGAGACTGGATGAA	TCACGGTAGCATAAGGTGCAGTTATGTATAGTACGCTCGC
6	CCCTCGTACACAATGGATAATCACGATGAGACTGGATGAA	TCACGGTAGCATAAGGTGCAAACTAGGTCGATCTGCCTGC
7	GACTCCCACTTCTTGTAATGTCACGATGAGACTGGATGAA	TCACGGTAGCATAAGGTGCACGGCACTGTAGTATAAACTC
8	CAGACGGTCAAATCCTCTAATCACGATGAGACTGGATGAA	TCACGGTAGCATAAGGTGCAAGTATCCCTAGTACGGCAAC
9	GAGGTAGCTCACTCCACATGTCACGATGAGACTGGATGAA	TCACGGTAGCATAAGGTGCAATGTCCGCTAATTCGCAGGC
10	GTCTAAGATCCTATCACACGTCACGATGAGACTGGATGAA	TCACGGTAGCATAAGGTGCAGGTGCCACTTACTAAACAAC
11	TTGCCACTGGTCCTGAATGGTCACGATGAGACTGGATGAA	TCACGGTAGCATAAGGTGCAAGTAAGCCACTTCAACGCAG
12	ATCAGGTCTGGAAGATTACGTCACGATGAGACTGGATGAA	TCACGGTAGCATAAGGTGCATAGTCCTCAGCATTATCAGC
13	AGCCTACTCTTCACGATTGTTCACGATGAGACTGGATGAA	TCACGGTAGCATAAGGTGCATAATGTCTGTGTAAGCCGCG
14	CTTTCAGAGGTCAGGTGTATTCACGATGAGACTGGATGAA	TCACGGTAGCATAAGGTGCAGGCCACAGGATTCTAAATAC
15	ACGACTCTAGCATGTCTACGTCACGATGAGACTGGATGAA	TCACGGTAGCATAAGGTGCAGGCCATATCACATTAACTGC
16		
17	CCCGCTAAGAGATATTGGTATCACGATGAGACTGGATGAA	TCACGGTAGCATAAGGTGCAATCACGTTCTGGAAGGGTTC
32	TGAGTATCACTTCCTCTACGTCACGATGAGACTGGATGAA	TCACGGTAGCATAAGGTGCAAGATGTCACCATATTCTCGC
35	GGCCCAAGTGTTAATTTGCTTCACGATGAGACTGGATGAA	TCACGGTAGCATAAGGTGCAGTATGAGAACTTCGCTACAG
38	GCCCAATGTCCAATAGCTTATCACGATGAGACTGGATGAA	TCACGGTAGCATAAGGTGCAGAGATCATACGTCCTACAAC
45	CGGCGCATCTGTTATCTAGTTCACGATGAGACTGGATGAA	TCACGGTAGCATAAGGTGCACAGTAACGATGTATCCACAG
51	GGCTCCCATGTTGGAATTATTCACGATGAGACTGGATGAA	TCACGGTAGCATAAGGTGCATAAGCTGATTGTCGCTACGG
53	CCTCGCTCGCAGTATAAATATCACGATGAGACTGGATGAA	TCACGGTAGCATAAGGTGCAGGCATAAGACTTCGTATCAG
57	CGAAGGCTAGTTCCAAATTGTCACGATGAGACTGGATGAA	TCACGGTAGCATAAGGTGCAATCTGATCCACTGCCGTATA

Primer sequences

Mux sequence	Rew qPCR	Frw qPCR	Rew Pre-AMP	Frw Pre-AMP
1	CGTGCTGCGAGAGTATTATCT	CCAGGTAGTAGTACGTCTGTT	CCGUUGTGCTGCGAGAGTATTA	CGCUUCAGGTAGTAGTACGTCT
2	CGTGAACCGTTATTTGGGTAC	CGGCCTCCTCCAATTAAAGAA	CGCUUGTGAACCGTTATTTGGG	CGCUUGGCCTCCTCCAATTAAA
3	CGTGACAGTGGCAGATATAAC	CGGATCACTCCAACTAGACTA	CGCUUGTGACAGTGGCAGATAT	CGCUUGGATCACTCCAACTAGA
4	CGAACTATGCTGACAGTACCG	CAGAGTCCACTTCCCATAATG	CGCUUGAACTATGCTGACAGTA	CGCUUAAGAGTCCACTTCCCATA
5	CGCGAGCGTACTATACATAAC	CCTACGACTAGGAGATAGATG	CCGUUGCGAGCGTACTATACAT	CGCUUCTACGACTAGGAGATAG
6	CGCAGGCAGATCGACCTAGTT	CCCCTCGTACACAATGGATAA	CGCUUGCAGGCAGATCGACCTA	CGCUUCCCTCGTACACAATGGA
7	CGAGTTTATACTACAGTGCCG	CGACTCCCACTTCTTGTAATG	CGCUUGAGTTTATACTACAGTG	CGCUUGACTCCCACTTCTTGTA
8	CGTTGCCGTACTAGGGATACT	CCAGACGGTCAAATCCTCTAA	CGCUUGTTGCCGTACTAGGGAT	CGCUUCAGACGGTCAAATCCTC
9	CGCCTGCGAATTAGCGGACAT	CGAGGTAGCTCACTCCACATG	CCGUUGCCTGCGAATTAGCGGA	CGCUUGAGGTAGCTCACTCCAC
10	CGTTGTTTAGTAAGTGGCACC	CGTCTAAGATCCTATCACACG	CGCUUGTTGTTTAGTAAGTGGC	CGCUUGTCTAAGATCCTATCAC
11	CCTGCGTTGAAGTGGCTTACT	CTTGCCACTGGTCCTGAATGG	CCGUUCTGCGTTGAAGTGGCTT	CGCUUTTGCCACTGGTCCTGAA
12	CGCTGATAATGCTGAGGACTA	CATCAGGTCTGGAAGATTACG	CGCUUGCTGATAATGCTGAGGA	CGCUUATCAGGTCTGGAAGATT
13	CCGCGGCTTACACAGACATTA	CAGCCTACTCTTCACGATTGT	CCGUUCGCGGCTTACACAGACA	CGCUUAGCCTACTCTTCACGAT
14	CGTATTTAGAATCCTGTGGCC	CCTTTCAGAGGTCAGGTGTAT	CGCUUGTATTTAGAATCCTGTG	CGCUUCTTTCAGAGGTCAGGTG
15	CGCAGTTAATGTGATATGGCC	CACGACTCTAGCATGTCTACG	CGCUUGCAGTTAATGTGATATG	CGCUUACGACTCTAGCATGTCT
16	CGAACTTCAGAACCGTGTTAC	CTATCTTAGGAGGAGCTTCTG	CGCUUGAACTTCAGAACCGTGT	CGCUUTATCTTAGGAGGAGCTT
17	CGAACCCTTCCAGAACGTGAT	CCCCGCTAAGAGATATTGGTA	CGCUUGAACCCTTCCAGAACGT	CGCUUCCCGCTAAGAGATATTG
32	CGCGAGAATATGGTGACATCT	CTGAGTATCACTTCCTCTACG	CCGUUGCGAGAATATGGTGACA	CGCUUTGAGTATCACTTCCTCT
35	CCTGTAGCGAAGTTCTCATAC	CGGCCCAAGTGTTAATTTGCT	CCGUUCTGTAGCGAAGTTCTCA	CGCUUGGCCCAAGTGTTAATTT
38	CGTTGTAGGACGTATGATCTC	CGCCCAATGTCCAATAGCTTA	CGCUUGTTGTAGGACGTATGAT	CGCUUGCCCAATGTCCAATAGC
45	CCTGTGGATACATCGTTACTG	CCGGCGCATCTGTTATCTAGT	CGCUUCTGTGGATACATCGTTA	CCGUUCGGCGCATCTGTTATCT
51	CCCGTAGCGACAATCAGCTTA	CGGCTCCCATGTTGGAATTAT	CCGUUCCGTAGCGACAATCAGC	CGCUUGGCTCCCATGTTGGAAT
53	CCTGATACGAAGTCTTATGCC	CCCTCGCTCGCAGTATAAATA	CGCUUCTGATACGAAGTCTTAT	CGCUUCCTCGCTCGCAGTATAA
57	CTATACGGCAGTGGATCAGAT	CCGAAGGCTAGTTCCAAATTG	CGCUUTATACGGCAGTGGATCA	CGCUUCGAAGGCTAGTTCCAAA

Hybridization oligonucleotide design

Mux sequence	Sequence as 3' oligo
1	GTGCTGCGAGAGTATTATCTTGCACCTTATGCTACCGTGACCTGCGAATCCAGTCT
2	GTGAACCGTTATTTGGGTACTGCACCTTATGCTACCGTGACCTGCGAATCCAGTCT
3	GTGACAGTGGCAGATATAACTGCACCTTATGCTACCGTGACCTGCGAATCCAGTCT
4	GAACTATGCTGACAGTACCGTGCACCTTATGCTACCGTGACCTGCGAATCCAGTCT
5	GCGAGCGTACTATACATAACTGCACCTTATGCTACCGTGACCTGCGAATCCAGTCT
6	GCAGGCAGATCGACCTAGTTTGCACCTTATGCTACCGTGACCTGCGAATCCAGTCT
7	GAGTTTATACTACAGTGCCGTGCACCTTATGCTACCGTGACCTGCGAATCCAGTCT
8	GTTGCCGTACTAGGGATACTTGCACCTTATGCTACCGTGACCTGCGAATCCAGTCT
9	GCCTGCGAATTAGCGGACATTGCACCTTATGCTACCGTGACCTGCGAATCCAGTCT
10	GTTGTTTAGTAAGTGGCACCTGCACCTTATGCTACCGTGACCTGCGAATCCAGTCT
11	CTGCGTTGAAGTGGCTTACTTGCACCTTATGCTACCGTGACCTGCGAATCCAGTCT
12	GCTGATAATGCTGAGGACTATGCACCTTATGCTACCGTGACCTGCGAATCCAGTCT
13	CGCGGCTTACACAGACATTATGCACCTTATGCTACCGTGACCTGCGAATCCAGTCT
14	GTATTTAGAATCCTGTGGCCTGCACCTTATGCTACCGTGACCTGCGAATCCAGTCT
15	GCAGTTAATGTGATATGGCCTGCACCTTATGCTACCGTGACCTGCGAATCCAGTCT
16	
17	GAACCCTTCCAGAACGTGATTGCACCTTATGCTACCGTGACCTGCGAATCCAGTCT
32	GCGAGAATATGGTGACATCTTGCACCTTATGCTACCGTGACCTGCGAATCCAGTCT
35	CTGTAGCGAAGTTCTCATACTGCACCTTATGCTACCGTGACCTGCGAATCCAGTCT
38	GTTGTAGGACGTATGATCTCTGCACCTTATGCTACCGTGACCTGCGAATCCAGTCT
45	CTGTGGATACATCGTTACTGTGCACCTTATGCTACCGTGACCTGCGAATCCAGTCT
51	CCGTAGCGACAATCAGCTTATGCACCTTATGCTACCGTGACCTGCGAATCCAGTCT
53	CTGATACGAAGTCTTATGCCTGCACCTTATGCTACCGTGACCTGCGAATCCAGTCT
57	TATACGGCAGTGGATCAGATTGCACCTTATGCTACCGTGACCTGCGAATCCAGTCT

addTable 3. Recovery (%) for nine of the PEA assays after technology transfer. Recovery is presented for all assays of which a commercial antigen was available.

Protein biomarker	Recovery (%)	Antigen supplier	Cat.no.
CEA	58-79	RnD Systems	4128-CM
TFRC	84-87	RnD Systems	2474-TR
MIF	58-78	RnD Systems	289-MF-002
OPN	62-84	RnD Systems	1433-OP
IGF-2	106-109	ProSpec*	cyt-265
NSE	95-107	ProSpec*	enz-324
IL8	11-129	RnD Systems	208-IL
TFF3	92-120	ProSpec*	cyt-005
s100A8	78-102	ProSpec*	pro-800

*PROSPEC Protein Specialities

addsTable 4 – Antigen mixes. Overview of the composition of the 10 different antigen mixes used for the assay specific test. Each mix is made from PBS + 0.1% BSA with spike-in of antigens in a concentration of 10 nM. Unspecific mix indicates that the assay specific antigen is not present in the mix, whereas specific mix indicates that the assay specific antigen is present in the mix.

TFRC unspecific mix (no. 1)		s100A8 unspecific mix (no. 2)	
Antigen	Conc. (nM)	Antigen	Conc. (nM)
TFRC	0	TFRC	10
IGF-2	10	IGF-2	10
TIMP-1	10	TIMP-1	10
S100A8	10	S100A8	0
TGF-b1	10	TGF-b1	10
**MIF unspecific mix (no.3)**		**TFF-3 unspecific mix (no.4)**	
Antigen	Conc. (nM)	Antigen	Conc. (nM)
MIF	0	MIF	10
OPN	10	OPN	0
TFF-3	10	TFF-3	10
NSE	10	NSE	10
CEA	10	CEA	10
IL-8	10	IL-8	10
**CEA unspecific mix (no. 5)**		**OPN unspecific mix (no. 6)**	
Antigen	Conc. (nM)	Antigen	Conc. (nM)
MIF	10	MIF	10
OPN	10	OPN	0
TFF-3	10	TFF-3	10
NSE	10	NSE	10
CEA	0	CEA	10
IL-8	10	IL-8	10
**IL-8 unspecific mix (no. 7)**		**NSE unspecific mix (no. 8)**	
Antigen	Conc. (nM)	Antigen	Conc. (nM)
MIF	10	MIF	10
OPN	10	OPN	10
TFF-3	10	TFF-3	10
NSE	10	NSE	10
CEA	10	CEA	0
IL-8	0	IL-8	10
**IL-8 unspecific mix (no. 9)**		**NSE unspecific mix (no. 10)**	
Antigen	Conc. (nM)	Antigen	Conc. (nM)
TFRC	10	TFRC	10
IGF-2	10	IGF-2	10
TIMP-1	0	TIMP-1	10
S100A8	10	S100A8	10
TGF-b1	10	TGF-b1	0

		Mix no.			Mix no.
TFRC	Specific 1	2	MIF	Specific 1	4
	Specific 2	9		Specific 2	5
	Specific 3	10		Specific 3	6
	Unspecific	1		Unspecific	3
s100A8	Specific 1	1	TFF-3	Specific 1	3
	Specific 2	9		Specific 2	5
	Specific 3	10		Specific 3	6
	Unspecific	2		Unspecific	4
CEA	Specific 1	3	IL-8	Specific 1	5
	Specific 2	4		Specific 2	6
	Specific 3	6		Specific 3	8
	Unspecific	5		Unspecific	7
OPN	Specific 1	4	NSE	Specific 1	5
	Specific 2	5		Specific 2	6
	Specific 3	7		Specific 3	7
	Unspecific	6		Unspecific	8

addTable 5 – Correlation Coefficients (R) between ELISA and PLA as well as ELISA and PEA. These results are calculated on the basis of ELISA, PLA and PEA analysis performed on both healthy individuals (n=70) and CRC patients (n=70) in order to complete an assessment of the newer techniques in comparison to the gold standard ELISA.

Spearman correlation values (R) for ELISA, PLA and PEA data			
**Protein biomarker (Panel no.)**	**PLA vs ELISA**	**PEA vs ELISA**	**PLA vs PEA**
CEA mAb (1)	0.90	0.98	0.91
CEA mAb (3)	0.95	0.96	0.73
CEA pAb (1)	0.59	0.80	0.47
CEA pAb (2)	0.34	0.59	0.30
IL-8 (1)	0.78	0.84	0.70
IL-8 (2)	0.82	0.73	0.60
TIMP-1 (3)	0.64	0.73	0.59
TIMP-1 (4)	0.53	0.75	0.52
CA 242 (2)	0.76	0.80	0.63
SLPI (3)	-0.62	-0.40	0.28
VEGF (1)	0.48	0.67	0.70

addTable 6. PLA- and PEA correlations. Presentation of the χ2 statistic for the PLA and PEA covariates from the multivariate logistic regression analysis for each biomarker, modeling the probability for colorectal cancer versus healthy individual for each marker. The χ2 value is largest for the PEA in all cases demonstrating that the PEA covariate is the best discriminator. The Pearson correlation coefficients demonstrate a substantial association between PLA and PEA levels.

	N = 140		
	**PLA (χ^2)**	**PEA (χ^2)**	**Pearson Correlation Coefficient, (R)**
**CEA**	0.09	4.45	0.84
**TFRC**	0.87	7.89	0.76
**NSE**	6.91	13.56	0.89
**CA19-9**	1.94	10.77	0.68
**DcR3**	0.01	2.26	0.78
**IL8**	2.32	6.93	0.52
**MIF**	2.73	12.54	0.68
**OPN**	3.34	15.40	0.53
**CA242**	0.001	8.39	0.73
**S100A8**	2.87	14.64	0.58
**TIMP-1**	2.72	14.74	0.70
**TFF3**	7.03	13.87	0.82

addTable 7 – Pearson correlation coefficients between the 12 potential CRC biomarkers, which were selected on a basis of a 0.01% discrimination. These numbers describe the association between markers. Markers with a strong correlation would likely not be independent in multivariate analysis.

Pearson Correlation Coefficients, (R's) N = 280												
	CEA	TFRC	NSE	CA19-9	DcR3	IL8	MIF	OPN	CA242	S100A8	TIMP-1	TFF3
CEA		0.20123	0.19181	0.31854	0.20352	0.23247	0.19131	0.24231	0.33619	0.22537	0.35350	0.41025
TFRC			0.10077	0.21173	0.31353	0.19751	0.05902	0.40044	0.21524	0.24929	0.35091	0.28781
NSE				0.17760	0.11241	0.32268	0.81213	0.21631	0.21594	0.24240	0.39015	0.22775
CA19-9					0.11325	0.19818	0.19947	0.13937	0.61707	0.21747	0.23762	0.20692
DcR3						0.41695	0.16487	0.24301	0.21049	0.22441	0.51373	0.40944
IL8							0.28564	0.14189	0.42582	0.18626	0.41143	0.33886
MIF								0.23503	0.17936	0.36859	0.42202	0.22968
OPN									0.07882	0.22151	0.50318	0.40693
CA242										0.22637	0.15761	0.23846
S100A8											0.37432	0.26398
TIMP-1												0.58855
TFF3												
